# Simulation of Heavy Metals Migration in Soil-Wheat System of Mining Area

**DOI:** 10.3390/ijerph16142550

**Published:** 2019-07-17

**Authors:** Aman Fang, Jihong Dong, Ru Zhang

**Affiliations:** 1 School of Environment Science and Spatial Informatics, China University of Mining and Technology, Xuzhou 221116, China; 2 Shaanxi Key Laboratory of Land Consolidation, Xi’an 710000, China

**Keywords:** soil-wheat system, heavy metals, concentration characteristic, risk assessment, migration model, mining area

## Abstract

Heavy metals in the soil of mining areas have become a primary source of pollution, which could cause deleterious health effects in people exposed through soil-plant systems via multi-pathways. A long-term field experiment under natural conditions was carried out to explore the distribution characteristic and migration law of heavy metals in a soil-wheat system of a mining area in Xuzhou. According to the second level standard of environmental quality standards for soils of China (GB 15618-1995), 30.8 g of CrCl_3_·6H_2_O, 8.3 g of Pb(CH_3_COO)_2_·3H_2_O, and 16.5 g of ZnSO_4_·7H_2_O were added into the soil of three experimental sites, respectively. The other experimental site with no additional compounds was used as the control site. The Cr, Pb, and Zn concentrations in the soil-wheat system were counted and their corresponding migration models were constructed. From 2014 to 2017, the mean concentrations of Cr (49.09 mg·kg^−1^), Pb (20.08 mg·kg^−1^), and Zn (39.11 mg·kg^−1^) in the soil of the addition sites were higher than that of the control site. The mean concentrations of Cr, Pb, and Zn in wheat of the addition sites were greater than that of the control site with the values of 3.29, 0.06, and 29 mg·kg^−1^. In comparison, the Cr, Pb, and Zn concentrations in the soil of all experimental sites were lower than the second level standard of environmental quality standards for soils of China (GB 15618-1995), whereas the Cr concentration exceeded its corresponding soil background value of Xuzhou in 2017. The Pb concentration in soil of the addition site was greater than its corresponding background value from 2014 to 2016. The Pb and Zn concentrations in wheat of all experimental sites were lower than the national hygienic standard for grains of China (GB2715-2005) and the national guidelines for cereals of China (NY 861-2004), but the Cr concentration significantly exceeded the national guidelines for cereals of China (NY 861-2004). By constructing the Identical-Discrepant-Contrary (IDC) gray connection models, the result showed that there was a non-linear relationship of Cr, Pb, and Zn concentrations in the soil-wheat system, and the absolute values of most correlation coefficients r were lower than 0.5 and the values of greyness $f_{G}\left(r\right)$ were more than 0.5. The curvilinear regression models could not reflect the relationship of Cr, Pb, and Zn concentrations in the soil-wheat system with the regression coefficient $r^{2}$ values far less than 1. Due to the values of regression coefficient $r^{2}$ being close to 1, this study suggested that the allocation estimation models could be used for simulating the Cr, Pb, and Zn migration in the soil-wheat system of a mining area in Xuzhou.

## 1. Introduction

The exploitation and utilization of mineral resources have changed the material cycle and energy flow of mining area ecosystem, resulting in serious ecological damage and environmental pollution. Accumulation of heavy metals in soil surrounding mining areas has aroused worldwide attention for their toxicity, non-biodegradability, and persistence [1,2,3]. The soil self-purification capacity can reduce the concentrations of heavy metals in soil [4]. When heavy metal concentrations exceed the maximum allowable limits of the soil environment, the soil will be polluted in varying degrees. Common heavy metals that have been identified in polluted soil include arsenic (As), cadmium (Cd), chromium (Cr), copper (Cu), mercury (Hg), lead (Pb), and zinc (Zn). Release of these metals without proper treatment poses a significant threat to public health because of their persistence, biomagnification, and accumulation in the food chain [5]. Heavy metals transferring from soil to food crops play an important role in leading to increased exposure of human beings to potentially toxic elements. 

Wheat, as an important staple agricultural crop worldwide, is at the beginning of the food chain and absorbs essential and non-essential elements from soil [6]. Previous studies have focused on the migration and accumulation of heavy metal in the soil-wheat system [7,8,9,10,11]. Common factors affecting the metal concentrations in wheat grain mainly include soil conditions, species, and geographic locations [12,13]. Stochastic models could simulate the migration of heavy metals in the soil of a large area, but the complex parameters and boundary conditions were often simplified based on personal experience, thus in neglecting the important external factors [14,15]. Deterministic models were always applied to analyse the heavy metal migration in the soil of a small area [16,17]. The above models were not suitable for simulating the migration of heavy metals in soils of mining areas with large area and complex environment. 

The migration law of heavy metals in a soil-crop system has been expressed by the mathematical model [18]. The multiple and stepwise regression models by considering these influencing factors have always been constructed to reflect the migration law [9,10,19,20,21,22]. A regression coefficient has been used to identify whether these models can reflect the linear relationship between heavy metal concentration and influencing factors. However, is there a linear relationship between metal concentration and each influencing factor? If not, is there a non-linear relationship or other relationship? Are there other optimal models to reflect these relationships? 

Xuzhou, as a mining city in China, has been experiencing 130 years of coal mining and processing. Coal resources are mainly distributed in Quanshan District, Tongshan District, Jiawang District, and Pei County and wheat is the main food crop. Existing research showed that the accumulation degrees of Cr, Pb, and Zn in the soil of these mining areas were severe, moderate, and mild, respectively [23,24,25,26]. Moreover, excessive intake of produce containing Cr may have stimulating, carcinogenic and mutagenic effects on the respiratory and gastrointestinal systems [27], high dietary intake of Pb may cause nervous system disorders and brain damage [28], and excessive intake of Zn may be a risk factor for high blood pressure and coronary heart disease [29]. Hence, this study selected the field experimental sites located in Quanshan District and designed four-phase experiments on the migration of heavy metals in a soil-wheat system under natural conditions. The aims were to (1) investigate the migration characteristics of Cr, Pb, and Zn in the soil-wheat system, (2) assess the pollution status of Cr, Pb, and Zn in the soil-wheat system, (3) analyse the relationship of Cr, Pb, and Zn concentrations in soil and wheat, and (4) construct the optimal model for simulating the heavy metal migration.

## 2. Materials and Methods 

### 2.1. Experimental Sites and Scheme 

The experimental sites are located in the remote sensing monitoring field of the Nanhu campus of China University of Mining and Technology in Quanshan District, Xuzhou City, Jiangsu Province (latitude 34°13′24″ North and Longitude 117°08′23″ East). It covers a total area of 47.2 × 11.8 m^2^, including the control site, Zn addition site, Pb addition site, and Cr addition site from west to east, respectively. Area per site is approximately 11.8 × 11.8 m^2^. The terrain of the experimental sites is flat while low in the north. The area is dominated by alluvial soils. A total of 10 soil samples (0–20 cm in depth) were randomly collected and tested to obtain the soil properties in June 2013 (Table 1). The soil pH was higher than 7.5.

Wheat sowing happened every October, and the concentration detection of heavy metals in soil and wheat samples occurred every June. The whole experimental period was divided into four phases (October 2013 to June 2014, October 2014 to June 2015, October 2015 to June 2016, October 2016 to June 2017). The specific experimental steps were as follows: (1) Layout of the sampling points. A total of 44 sampling points were selected at the experimental sites, with 11 sampling points at each site (one of them was a supplemental point). The sampling points at the control site and Cr addition site adopted the plum blossom pattern. There were two closed dry wells located at the Pb addition site and Zn addition site, respectively. In order to reduce the influence of closed dry wells, the sampling points at these two sites were arranged in a chessboard pattern and snake pattern, respectively. (2) Bury of the organic glass columns. Unsealed organic glass columns (20 cm in height and 20 cm in diameter) at both ends were inserted vertically into the soil at each sampling point, keeping their upper ends flush with the soil. (3) Addition of related compounds. Quanshan District is dominated by farmland, and the soil quality is critical to food security and human health. Hence, the additive amount of heavy metals was based on the second level standard of environmental quality standards for soils of China (GB 15618-1995) which always used for the soils of farmland, vegetable field, and tea plantation [30]. CrCl_3_·6H_2_O, Pb(CH_3_COO)_2_·3H_2_O, and ZnSO_4_·7H_2_O were weighed at 30.8 g, 8.3 g, and 16.5 g (± 0.01 g), respectively. Nylon bags (size: 10 × 15 cm^2^; density: 100 mesh; material: polyethylene) with those compounds were placed at the bottom of the organic glass columns and buried in soil. (4) Wheat sowing. From 2013 to 2016, the winter wheat was planted at the experimental sites in October each year. There were 13 or 15 winter wheat grains of the same type in each organic glass column. During the period from sowing to ripening, we observed and recorded the growth characteristics of wheat every 5–7 days.

### 2.2. Samples Collection and Chemical Analysis

Wheat (grain part) and soil (0–20 cm in depth) samples were collected from the 44 sampling points of the control site, Cr addition site, Pb addition site, and Zn addition site in June each year. Meanwhile, a total of 10 soil samples (0–20 cm in depth) were randomly collected to obtain soil pH. All soil samples at the experimental sites were air-dried at room temperature, and then passed through a 0.154 mm nylon sieve. A 0.2 g (± 0.0001 g) dried and powdered soil sample was digested using a mixture of concentrated acids with 5 mL of 90% (w/w) HCl and 5 mL of 90% (w/w) HNO_3_ at a temperature of 105 °C for 8 h [18]. All wheat samples were washed with distilled water and oven-dried at 65 °C for 48–72 h. The dried wheat samples were crushed separately through a steel grinder and the crushed material was passed through a 0.154 mm nylon sieve. A 0.5 g (± 0.0001 g) powdered wheat sample was digested using a mixture with 3mL of 90% (w/w) H_2_O_2_ and 5 mL of 90% (w/w) HNO_3_ at a temperature of 105 °C for 6 h [31,32]. The Cr, Pb, and Zn concentrations in soil and wheat samples were determined by inductively coupled plasma-mass spectrometry (ICP-MS, Agilent, Palo Alto, CA, USA). The soil pH was determined at the soil: water ratio of 1: 2.5 at room temperature by using a combined glass-calomel electrode (PHS-3E, Rex, Shanghai INESA Scientific Instrument Co., Ltd., Shanghai, China) [33].

### 2.3. Data Processing

The mean value and standard deviation were calculated with Microsoft Excel (Version 14, Microsoft, Inc., Redmond, Washington, DC, USA). The two-way analysis of variance (two-way ANOVA), the change rate, and the construction of identical-discrepant-contrary gray connection model, curvilinear regression model and allocation estimation model were achieved by using IBM SPSS statistics software (Version 19.0, Armonk, NY, USA). The spatial distribution of Cr, Pb, and Zn concentrations in soil was performed using ArcGIS software (Version 10.2, ESRI, Inc., Redlands, CA, USA). 

Bioconcentration factor (BCF) can reflect the transferability of heavy metals from soil to crop (e.g., wheat, corn, rice) [34]. BCF is expressed as the following equation:

(1) $$BCF=C_{w}/C_{S}$$

Where $C_{w}$ is the measured concentration of heavy metal in wheat; and Cs is the measured concentration of heavy metal in soil. In this study, BCF was performed with OriginPro software (version 8.5, Origin Lab Ltd., Northampton, MA, USA).

The ecological risk index, which is based on both metal concentrations and its toxicity, was used to assess the ecological risks posed by heavy metals in soil [35]. $E_{r}^{i}$ represents the ecological risk index of each individual toxic metal in soil. It is calculated using the following formulas:

(2) $$E_{r}^{i}=T_{r}^{i}\times C^{i}/C_{n}^{i}$$

Where $T_{r}^{i}$ is the toxic coefficient of soil heavy metal *i*. Based on previously published research, the toxic coefficients of Cr, Pb, and Zn were 2, 5, and 1, respectively [34]; $C_{i}$ is the measured concentration of heavy metal in soil; $C_{n}^{i}$ is the background value of heavy metal *i* in soil of Xuzhou City [23]. Five categories of pollution are distinguished: $E_{r}^{i}<40$ (low), $40\leq E_{r}^{i}<80$ (moderate), $80\leq E_{r}^{i}<160$ (considerable), $160\leq E_{r}^{i}<320$ (high), and $E_{r}^{i}\geq 320$ (very high) [36].

(3) $$P_{i}=C_{w}/C_{o}$$

Where $P_{i}$ is the pollution index of heavy metal *i* in wheat; $C_{w}$ is the measured concentration of heavy metal in wheat; $C_{o}$ is the limited values of the national hygienic standard for grains of China (GB2715-2005) [37] and the national guidelines for cereals of China (NY 861-2004) [38]. The risk degree is classified into five categories: $P_{i}\leq 0.7$ (unpolluted), $0.7<I_{i}\leq 1.0$ (warning limit), $1.0<I_{i}\leq 2.0$ (low polluted), $2.0<I_{i}\leq 3.0$ (moderately polluted), and $I_{i}>3.0$ (strongly polluted) [39].

## 3. Results and Discussion

### 3.1. Concentration Characteristics of Cr, Pb, and Zn in Soil-Wheat System

Table 2 shows the statistical characteristics of Cr, Pb, and Zn in the soil−wheat system of the experimental sites during the whole experimental period. From 2014 to 2017, for the addition sites, the mean concentrations of Cr, Pb, and Zn in soil were 49.09, 20.08, and 39.11 mg·kg^−1^, and in wheat were 3.29, 0.06 and 29 mg·kg^−1^, respectively. For the control site, the mean concentrations of Cr, Pb, and Zn in soil were 47.66, 13.81, and 37.26 mg·kg^−1^, and in wheat were 2.89, 0.05 and 23.94 mg·kg^−1^, respectively. In terms of the soil of the addition sites, the Cr concentration first decreased by 4.21 mg·kg^−1^ from 45.22 mg·kg^−1^ in 2014 to 41.01 mg·kg^−1^ in 2015, followed by a continuous increase to 62.43 mg·kg^−1^ in 2017. The Zn concentration tended to decrease from 2014 to 2015, but increased to 46.31 mg·kg^−1^ in 2017. The Pb concentration sharply increased to 27.22 mg·kg^−1^ between 2014 and 2015, but steeply decreased to 12.23 mg·kg^−1^ in the next two years. It stated that there was an obvious migration phenomenon of Cr and Zn in soil of the addition sites in 2015. However, Pb was accumulated in soil of the additional site in 2015. In terms of the soil of the control site, between 2014 and 2017, there was a sustained growth trend of the Cr and Pb concentrations, which might be caused by the factors (e.g., atmospheric dustfall, organic matter, and soil pH). The variation tendency of Zn concentration was the same as that of the Zn addition site. For the soil of all experimental sites, the Zn concentration did not exceed the second level standard of environmental quality standards for soils of China (GB 15618-1995) and its corresponding background value of Xuzhou City. However, the Cr concentration was higher than its corresponding background value in 2017. As for the soil of the addition site, the Pb concentration was greater when compared with its corresponding background value from 2014 to 2016.

The variation tendency of Cr, Pb, and Zn concentrations in wheat was consistent with that in soil. In terms of the wheat of the addition sites, the Cr concentration decreased to 1.72 mg kg^−1^ in 2015, but increased to 4.33 mg·kg^−1^ in 2017. The Pb concentration exhibited an unstable tendency during the whole experimental period. The Zn concentration experienced a trend of first decreasing and then increasing from 2014 to 2017. For the wheat of the control site, the variation tendency of Cr and Pb concentrations were the same as that of the addition sites. In terms of the wheat of all experimental sites, the Pb and Zn concentrations were observed to be lower than the national hygienic standard for grains of China (GB 2715-2005) and the national guidelines for cereals of China (NY 861-2004), but the Cr concentration significantly exceeded the national guidelines for cereals of China (NY 861-2004). 

Most of the Cr, Pb, and Zn concentration differences were more than 0, illustrating that these heavy metal concentrations in the soil-wheat system of the addition sites were higher than that of the control site. It also explains that the anthropogenic activity of adding heavy metals promotes the transformation and migration of heavy metals into the soil-wheat system directly or indirectly. Meanwhile, the heavy metal concentrations in soil of the addition sites approached that of the control site with the differences gradually decreasing. The Cr concentration in soil of the addition site was closest to that of the control site in 2016. The Pb and Zn concentrations in soil of the addition sites were close enough to that of the control site in 2017. 

Table 2 also presents the results of two-way ANOVA for the heavy metal concentrations in soil-wheat system of all experimental sites during the experimental period. The concentrations of Cr, Pb, and Zn were significantly different between the experimental phases (*P* < 0.05), but no significant difference was observed between the experimental sites in terms of the analysed metals (*P* > 0.05) except for Pb in soil. Factors, such as soil properties, atmospheric dust, rainfall, and industrial pollution, may cause the migration and accumulation of metals in soil-wheat system [40,41]. Due to there is no industrial and agricultural waste around the experimental sites, the metal migration may be caused by soil properties, rainfall, and atmospheric dustfall. Compared with 2015, the dust concentration in Xuzhou City increased by 310.9% from January to August 2016 [42]. The environmental status bulletin of Xuzhou City showed that the average annual concentration of inhalable particles PM10 was 0.74 times over the standard and the exceeding standard rate of daily average concentration was 29.6% in 2016 [43]. Also, existing research stated that the atmospheric dustfall in Xuzhou City might be an important source of heavy metals (e.g., Cd, Cr, Pb, and Cu) in soil-wheat system [44,45].

### 3.2. Change Rates of Cr, Pb, and Zn Concentrations in the Soil-Wheat System

**Figure 1:**
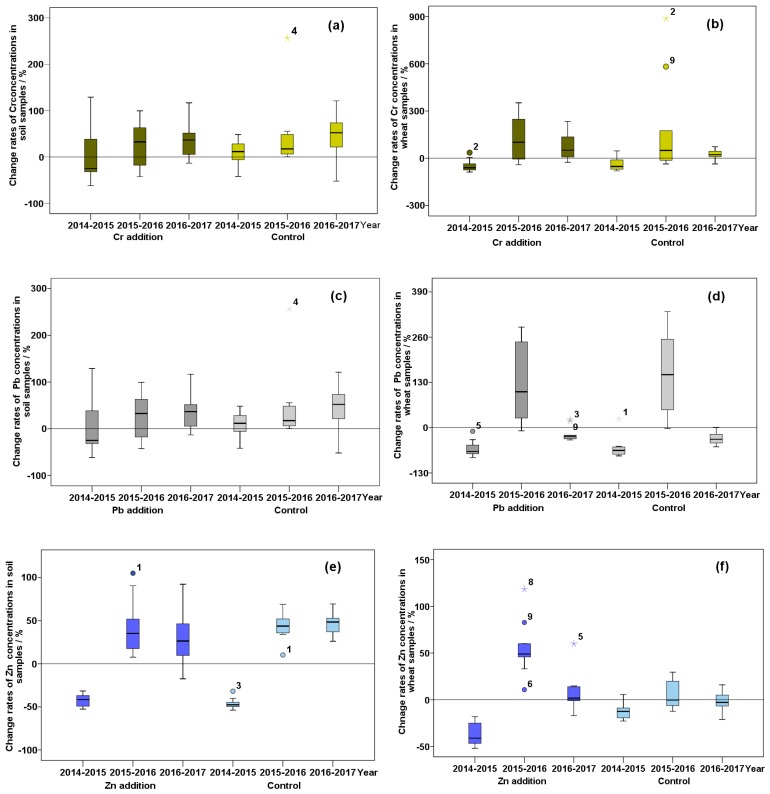
Change rates of Cr, Pb, and Zn concentrations in the soil-wheat system from 2014 to 2015, 2015 to 2016, and 2016 to 2017: (**a**) Cr in soil; (**b**) Cr in wheat; (**c**) Pb in soil; (**d**) Pb in wheat; (**e**) Zn in soil; and (**f**) Zn in wheat. For the addition sites and control site, Cr concentrations are expressed by dark yellow and light yellow, Pb concentrations are expressed by dark gray and light gray, and Zn concentrations are expressed by dark blue and light blue. Circles and asterisks with numbers are samples with outliers.

Figure 1 exhibits the change rates of Cr, Pb, and Zn concentrations in soil-wheat system during the experimental period. In general, from 2014 to 2015, 2015 to 2016, and 2016 to 2017, the change rates of Cr, Pb, and Zn concentrations ranged from −50% to 150%, −50% to 270%, and −50% to 90% in soil, and −100% to 400%, −70% to 340%, and −70% to 70% in wheat. The change rate of Cr concentration in soil was consistent with that in wheat. The medians of Cr concentration change rates in soil and wheat samples were less than 0 from 2014 to 2015, illustrating that Cr concentrations in more than half of the soil and wheat samples tended to decrease. The Cr concentrations in approximately 75% of the soil and wheat samples showed an increasing trend with the lower quartiles of their change rates greater than 0 during the next two years (2015–2017). The significant differences were found in the change rates of Pb concentration between soil and wheat samples. Between 2014 and 2015, the Pb concentrations in all the soil samples increased with their change rates greater than 0, but in all the wheat samples decreased with their change rates less than 0. There was a decreasing trend of the Pb concentrations in more than 50% soil samples, but an increasing trend was found in over 75% wheat samples between 2015 and 2016. From 2016 to 2017, there were more than 75% soil samples at which the Pb concentrations tended to increase, yet the Pb concentrations in all wheat samples showed a decreasing trend. Due to the change rates being lower than 0, the Zn concentrations in all soil and wheat samples presented a downward trend (2014–2015). More than 50% of soil and wheat samples appeared a tendency to increase (2015–2017).

### 3.3. Migration Distributions of Cr, Pb, and Zn Concentrations in the Soil-Wheat System

**Figure 2:**
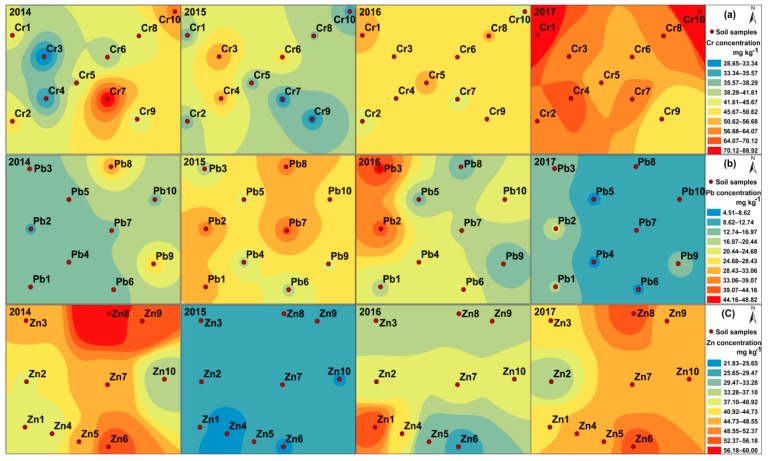
Spatial distributions of heavy metal concentrations in soil from 2014 to 2017: (**a**) Cr addition site; (**b**) Pb addition site; and (**c**) Zn addition site.

In general, the farther away from the pollution source, the lower concentrations of heavy metals in soil. The spatial distribution characteristic of heavy metal concentration was analysed by using the method of inverse distance to a power in ArcGIS software. Figure 2 exhibits the spatial distributions of Cr, Pb, and Zn concentrations in soil of the addition sites. In terms of the Cr concentration, the value of No. 7 was the highest, and No. 3 was the lowest in 2014. The highest value was No. 3 in 2015. The value of No.1 exceeded that of other samples in 2016 and 2017. The Cr concentrations in soil samples located in the northwest of the additional site showed a significant increase during the experimental period. For the Pb concentration in soil, the highest value was No. 8 in 2014. The value of No. 7 was greater than that of other samples in 2015. The values of No. 3 and No. 2 were the highest in 2016 and 2017, respectively. There was an increasing trend of the Pb concentrations in soil samples situated in the west of the addition site from 2014 to 2016. In terms of the Zn concentration in soil, No. 8 appeared the highest value in 2014. The value of No. 1 was higher than that of other samples in 2016. There was no obvious variation law in the spatial distribution of Zn concentration in soil. The reason for significant differences in the spatial distribution of Cr and Pb concentrations in soil may be related to the terrain of the experimental site. The lower terrain area in the north and west of the Cr and Pb addition sites may influence the rainwater gathering, affecting the migration of heavy metal in soil. The wheat may contribute to the migration and transformation of heavy metal in soil by its enrichment capacity. Also, a highway is located in the north of the experimental sites, and the vehicle exhaust may be a potential resource.

**Figure 3:**
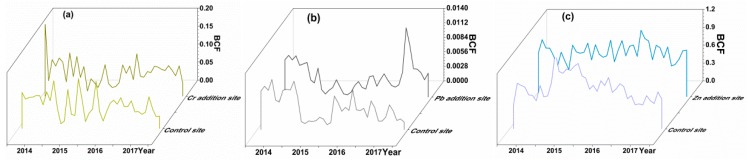
Bioconcentration factor (BCF) of heavy metal in wheat from 2014 to 2017: (**a**) Cr; (**b**) Pb; and (**c**) Zn.

Figure 3 exhibits the ratio of Cr, Pb, and Zn concentrations in wheat to those in soil (BCF) of the experimental sites from 2014 to 2017. BCF of Cr, Pb, and Zn ranged from 0.01 to 0.2, 0.001 to 0.014, and 0.03 to 1.2, respectively, indicating that wheat had a different accumulation capacity for these metals. BCF of these metals was in the order of Zn > Cr > Pb, implying the high transferability of Zn from soil to wheat. The high BCF of Zn in wheat may be explained by a combination of factors, involving the concentration of Zn in soil, the Zn chemistry, and soil properties [26,46]. The BCF of Pb ranged from 0.001 to 0.014, indicating that Pb had a lower transferability from soil to wheat than those of Zn and Cr. From 2014 to 2017, the soil pH average values of the experimental sites were 7.83, 7.74, 7.79, and 7.61. The transfer capacity of Pb is likely to be associated with the value of soil pH (above 7.5) [47].

### 3.4. Risk Index of Cr, Pb, and Zn in Soil-Wheat System

Table 3 shows the risk indices of Cr, Pb, and Zn in soil and wheat of the experimental sites. From 2014 to 2017, the $E_{r}^{i}$ values of Cr, Pb, and Zn in soil were less than 40, presenting a low ecological risk level. There was no pollution of Pb and Zn in wheat with their $P_{i}$ values lower than 0.7. However, the $P_{i}$ value of Cr in wheat ranged from 1.72 to 4.33, which appeared low, moderately, and strongly pollution levels.

### 3.5. Identical-Discrepant-Contrary (IDC) Gray Connection Model

The correlation of two sets (e.g., identity, difference, and opposition) can be obtained by the analysis of the Identical-Discrepant-Contrary (IDC) trend. The division method of the IDC trend is critical to the accuracy of the set partition result. According to the existing research foundation, there was relatively high accuracy by using the average-division iterative method to divide the IDC trend [48]. Therefore, the average-division iterative method was selected to analyse the correlation of heavy metals in soil and wheat.

$x_{1},x_{2},\;\cdots ,\;x_{n}$ are defined as the independent variable x, and $y_{1},y_{2},\;\cdots ,\;y_{n}$ are defined as the dependent variable y. Previous research showed that multi-subintervals could more accurately describe the IDC trend of the data set [48]. The variables x and y are equally divided into six subintervals, respectively. The six subintervals are set up to A-, A, B-, B, C-, and C in turn. Comparing the set (x, y) to be divided with the equalized subintervals, we obtain the letter combination of A-AB-BC-C. The set (x, y) are divided based on the IDC trend standard [48]. According to the division result, the number of identical, discrepant, and contrary is counted and recorded as $s_{1}$, $f_{1}$, and $p_{1}$, respectively. Meanwhile, the set pairs of identity, opposition, and extreme value are separated. The remaining variables x and y are again equally divided into six subintervals, respectively, and the remaining set (x, y) are divided based on the IDC trend standard. The number of identical, discrepant and contrary is counted and recorded as $s_{2}$, $f_{2}$, and $p_{2}$, respectively. The partition result is obtained until the set pairs are completely separated. In general, the partition result is identical after three iterations. The total number of identical, discrepant and contrary is counted as s, f, and p, respectively. 

$f_{G}\left(r\right)$ refers to the grey degree of the correlation coefficient r of variables x and y. In other words, it means the uncertainty degree of the correlation of variables x and y. The smaller the $\;f_{G}\left(r\right)$ value, the lower the uncertainty degree of the correlation between variables x and y. This suggests that the value of correlation coefficient r is trusted.

(4) $$f_{G}\left(r\right)=1-\frac{S}{N},\;r>0;\;f_{G}\left(r\right)=1-\frac{P}{N},\;r<0$$

Where r represents the correlation coefficient of variables x and y, and N represents the sum of s, f, and p. 

Firstly, the Cr, Pb, and Zn concentrations in soil from 2014 to 2017 were defined as the independent variable x, and the Cr, Pb, and Zn concentrations in wheat from 2014 to 2017 were defined as the dependent variable y. The set (x, y) was divided based on the IDC trend standard. According to the number of identity, difference, and opposition, we counted the values of s, f, and p. Secondly, the relationship of Cr, Pb, and Zn in soil and wheat was analysed and the correlation coefficient r was calculated. Thirdly, the grey degree $f_{G}\left(r\right)$ was calculated based on Equation (4). Table 4 shows the correlation coefficients and greyness of Cr, Pb, and Zn in soil and wheat of the experimental sites from 2014 to 2017. The absolute values of most correlation coefficient r were lower than 0.5, and their greyness values were more than 0.5, indicating that there was a non-linear relationship of the Cr, Pb, and Zn concentrations in soil and wheat.

### 3.6. Curvilinear Regression Model

As a commonly used statistical method for the relationship analysis between heavy metal concentrations in soil-crop system, the curvilinear regression model is constructed based on the known independent variable x and dependent variable y, and used for explaining the curvilinear relationship between these variables [49,50]. The conceptual model is shown in formula (5):

(5) y=f(x,β)+ε

where x represents the observable independent random variable, β represents the parameter vector to be estimated, y represents the independent observation variable, and its average value depends on x and β, and ε represents the random error. The common curvilinear regression model includes the quadratic curve model, composite model, growth model, logarithm model, S-shaped model, parabola model, reciprocal model, power function model, and logistic model.

The Cr, Pb, and Zn concentrations in soil from 2014 to 2017 were defined as the independent variable x, and the Cr, Pb, and Zn concentrations in wheat from 2014 to 2017 were defined as the dependent variable y. The curvilinear regression analysis of Cr, Pb, and Zn concentrations in soil and wheat was done by using IBM SPSS Statistics 19. For each heavy metal, the optimal curvilinear regression model was selected under considering the regression coefficient and significance test. Successfully, the optimal curvilinear regression models were obtained for Cr ($r^{2}=0.077$, P<0.001), Pb ($r^{2}=0.126$, P<0.001), and Zn ($r^{2}=0.352$, P<0.001) (Table 5). The $r^{2}$ values were far less than 1, indicating that there was a low fitting accuracy of these models.

### 3.7. Allocation Estimation Model

The absorption of pollutants by crop in the soil environment depends on the allocation relationship between soil and crop, which varies with the pollutant concentration in soil and the crop species [51]. The formula for allocation estimation model of heavy metal pollutants in the soil-wheat system can be expressed as follows:

(6) Cw=fws×Cs

Where Cw represents the measured concentration of heavy metal in wheat; Cs represents the measured concentration of heavy metal in soil; and fws represents the allocation coefficient of heavy metal concentration in soil-wheat system. In this study, BCF was the allocation coefficient fws.

The bioconcentration factor (BCF) of Cr, Pb, and Zn in wheat from 2014 to 2017 of the experimental sites was calculated in accordance with the formula (1). To assess the relationship between the bioaccumulation capacity of wheat and the heavy metal concentration in soil, the relational models were employed and selected under considering the regression coefficient and significance test. The optimal relational models were obtained for Cr ($r^{2}=0.627$, P<0.001), Pb ($r^{2}=0.632$, P<0.001), and Zn ($r^{2}=0.784$, P<0.001) (Table 6). The fitting accuracies of selected optimal models were high with their $r^{2}$ values close to 1. Also, we finally explored the corresponding allocation estimation models by combining the relational models with the formula (6) (Table 6).

### 3.8. Selection and Verification of Optimal Model

In the case of the theory, the curvilinear regression model was constructed to analyse the possible curvilinear relationships between the heavy metal concentrations in soil and wheat. The allocation estimation model was established in consideration of the allocation principle of heavy metals in the soil-wheat system. In the case of the fitting accuracy, the $r^{2}$ values of the allocation estimation models were higher than that of the curvilinear regression models. Therefore, the allocation estimation models were selected to simulate the migration of heavy metals in the soil-wheat system with high-fitting precision. 

**Figure 4:**
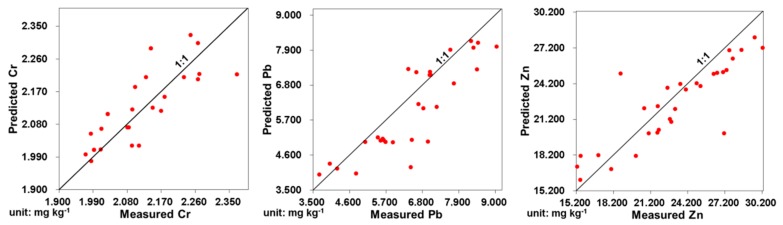
Predicted and measured values of Cr, Pb, and Zn concentrations in wheat in Liuxin mining area.

The Liuxin mining area is located in Liuxin town, Xuzhou City, Jiangsu Province (latitude 34°21′38″ North and Longitude 117°08′29″ East). We randomly selected 30 sampling points in the mining area. The soil and wheat samples were collected and the Cr, Pb, and Zn concentrations in all samples were detected in July 2012. According to the selected allocation estimation models, the predicted values of Cr, Pb, and Zn in wheat were calculated based on their measured values in soil. Figure 4 exhibits the dispersed point charts between measured and predicted values of Cr, Pb, and Zn in wheat. The dispersed points of Cr, Pb, and Zn were close to the 1:1 trend line, indicating that better prediction results were obtained with these allocation estimation models. The relative standard errors of Cr, Pb, and Zn suggested that there were more accurate prediction results (Table 7).

## 4. Conclusions

The Cr and Zn concentrations in soil-wheat system of the experimental sites showed a decrease, followed by an increase, and the Pb concentration exhibited an unstable tendency through the whole experimental period. The Cr, Pb, and Zn concentrations in the soil-wheat system of the addition sites were higher than that of the control site, explaining that the anthropogenic activity of adding heavy metals promotes the transformation and migration of heavy metals into the soil-wheat system directly or indirectly. There was a low ecological risk of Cr, Pb, and Zn in the soil of the experimental sites. Cr showed low, moderately, and strongly pollution risks in wheat of the experimental sites. There was a non-linear relationship of Cr, Pb, and Zn concentrations in soil and wheat by the analysis of the Identical-Discrepant-Contrary trend. Compared with curvilinear regression models, allocation estimation models could better realize the simulation of Cr, Pb, and Zn migration in soil-wheat system with the high values of regression coefficients. In this study, we initially realized the simulation of heavy metal migration by analysing the concentration characteristic and constructing the allocation estimation models. We will explore and collect the related factors to improve the experiment in the future.

## Figures and Tables

**Table 1:** Mean and standard deviation (SD) (mean ± SD) of soil properties of the experimental sites in 2013.

Soil Samples	Bulk Density g (cm^3^)^−1^	Porosity %	Moisture Content %	Organic Matter mg·kg^−1^	pH	Heavy Metal Concentrations mg·kg^−1^		
						Cr	Pb	Zn
*n* = 10	1.23 ± 0.08	48.74 ± 0.33	20.33 ± 0.41	12.18 ± 0.73	7.89 ± 0.12	33.58 ± 0.52	8.74 ± 0.65	26.74 ± 1.58

**Table 2:** Mean and standard deviation (SD) (mean ± SD) of heavy metal concentrations in soil and wheat from 2014 to 2017.

Type	Heavy Metal	Site	Year				Two-Way Analysis of Variance (ANOVA)				MAC-II^a^	BV ^b^	LC ^c^
							*F* value		*P* value				
			2014	2015	2016	2017							
							Years	Sites	Years	Sites			
Soil mg·kg^−1^	Cr	addition	45.22 ± 12.13	41.01 ± 14.56	47.68 ± 7.99	62.43 ± 10.68	15.262	0.209	<0.05	0.649	350	55.50	-
		control	34.96 ± 7.33	36.30 ± 5.18	51.46 ± 24.96	67.92 ± 12.83							-
	Pb	addition	17.14 ± 9.19	27.22 ± 11.90	23.73 ± 9.96	12.23 ± 6.73	5.142	14.660	<0.05	<0.05	350	16.30	-
		control	9.80 ± 1.11	14.69 ± 1.22	14.73 ± 3.05	16.00 ± 2.01							-
	Zn	addition	46.45 ± 7.15	26.33 ± 1.94	37.33 ± 6.81	46.31 ± 5.29	84.804	3.167	<0.05	0.079	300	91.10	-
		control	46.32 ± 5.74	23.26 ± 2.31	33.12 ± 2.30	46.34 ± 2.82							-
	Cr addition-control ^d^		7.26 ± 3.56	4.71 ± 1.48	−3.78 ± 1.33	−5.49 ± 1.19	-	-	-	-		-	-
	Pb addition-control ^e^		7.33 ± 2.31	12.53 ± 4.28	9.00 ± 2.48	−3.77 ± 0.59	-	-	-	-		-	-
	Zn addition-control ^f^		8.37 ± 2.73	3.07 ± 1.59	4.21 ± 1.40	−0.03 ± 0.07	-	-	-	-		-	-
Wheat mg·kg^−1^	Cr	addition	4.16 ± 1.99	1.72 ± 0.85	2.98 ± 1.14	4.33 ± 0.69	7.718	2.116	<0.05	0.999	-	-	1.0
		control	3.14 ± 0.55	1.96 ± 1.30	3.06 ± 1.19	3.42 ± 0.69							
	Pb	addition	0.09 ± 0.01	0.03 ± 0.02	0.06 ± 0.01	0.05 ± 0.01	35.978	3.462	<0.05	0.067	-	-	0.2
		control	0.07 ± 0.01	0.03 ± 0.02	0.06 ± 0.02	0.04 ± 0.02							
	Zn	addition	32.63 ± 4.26	20.03 ± 1.81	30.98 ± 5.16	32.37 ± 4.60	18.294	38.520	<0.05	0.032	-	-	50
		control	25.78 ± 2.68	22.69 ± 1.81	23.76 ± 2.47	23.51 ± 3.20							
	Cr addition-control ^g^		1.02 ± 0.38	−0.24 ± 0.23	−0.08 ± 0.01	0.91 ± 0.07	-	-	-	-	-	-	-
	Pb addition-control ^h^		0.02 ± 0.01	0.00 ± 0.02	0.00 ± 0.01	0.01 ± 0.02	-	-	-	-	-	-	-
	Zn addition-control ^i^		6.85 ± 2.26	−2.66 ± 1.05	7.22 ± 2.84	8.85 ± 3.95	-	-	-	-	-	-	-

Note: - no giving. ^a^ Maximum allowable concentration-II, the second level standard of environmental quality standards for soils regulated by the State Environmental Protection Administration of China (GB 15618-1995); unit: mg·kg^−1^. ^b^ Heavy metal background values in soils of Xuzhou City, China [23]; unit: mg·kg^−1^. ^c^ Limited concentration, the national hygienic standard for grains (GB 2715-2005) and the national guidelines for cereals (NY 861-2004). unit: mg·kg^−1^. ^d, e, f^ Differences of soil Cr, Pb and Zn concentrations between addition sites and control site, respectively; unit: mg·kg^−1^. ^g, h, i^ Differences of wheat Cr, Pb and Zn concentrations between addition sites and control site, respectively; unit: mg·kg^−1^.

**Table 3:** Mean and standard deviation (SD) (mean ± SD) of risk indices of Cr, Zn and Pb in soil and wheat.

Type	Heavy Metal	Site	Year			
			2014	2015	2016	2017
Soil	Cr	addition	1.63 ± 0.44	1.48 ± 0.52	1.72 ±0.29	2.25 ± 0.39
		control	1.26 ± 0.26	1.31 ± 0.19	1.85 ± 0.89	2.45 ± 0.46
	Pb	addition	5.26± 1.41	8.35 ± 1.65	7.28 ± 2.06	3.75 ± 1.06
		control	3.01± 0.34	4.51 ± 0.38	4.52 ± 0.93	4.91 ± 0.62
	Zn	addition	0.93± 0.08	0.53 ± 0.02	0.75 ± 0.08	0.93 ± 0.06
		control	0.87 ± 0.06	0.47 ± 0.03	0.66 ± 0.03	0.97 ± 0.04
Wheat	Cr	addition	4.16 ± 1.99	1.72 ± 0.85	2.98 ± 1.14	4.33 ± 0.69
		control	3.14 ± 0.55	1.96 ± 1.30	3.06 ± 1.19	3.42 ± 0.69
	Pb	addition	0.45± 0.07	0.17 ± 0.08	0.32 ± 0.07	0.26 ± 0.07
		control	0.37± 0.03	0.15 ± 0.08	0.32 ± 0.08	0.22 ± 0.08
	Zn	addition	0.65± 0.08	0.40 ± 0.04	0.62 ± 0.10	0.65 ± 0.09
		control	0.52 ± 0.51	0.45 ± 0.32	0.48 ± 0.49	0.47 ± 0. 61

**Table 4:** The correlation coefficient and greyness of Cr, Pb, and Zn concentrations in soil and wheat.

Site	Heavy Metal	2014		2015		2016		2017	
		*r*	*f_G_* (*r*)	*r*	*f_G_* (*r*)	*r*	*f_G_* (*r*)	*r*	*f_G_* (*r*)
Cr addition	Cr	−0.276	0.71	0.166	0.62	−0.084	0.75	0.031	0.44
Pb addition	Pb	0.708	0.25	−0.065	0.50	0.370	0.60	−0.056	0.51
Zn addition	Zn	−0.226	0.79	−0.272	0.67	0.296	0.73	0.021	0.77
Control	Cr	0.415	0.27	−0.250	0.69	−0.059	0.82	−0.045	0.58
	Pb	−0.410	0.58	−0.059	0.34	−0.214	0.64	−0.258	0.49
	Zn	−0.425	0.80	−0.120	0.91	0.325	0.36	0.019	0.53

**Table 5:** The optimal curvilinear regression models of the Cr, Pb and Zn concentrations in the soil-wheat system.

Model	*r* ^2^	Significance
$y_{Cr}=3E-05x_{Cr}{}^{3}-0.0064x_{Cr}{}^{2}+0.4345x_{Cr}-5.8682$	0.077	P<0.001
$y_{Pb}=7E-06x_{Pb}{}^{3}+0.0006x_{Pb}{}^{2}-0.0153x_{Pb}+0.1717$	0.126	P<0.001
$y_{Zn}=-0.0004x_{Zn}{}^{3}+0.0371x_{Zn}{}^{2}-0.618x_{Zn}+20.903$	0.352	P<0.001

**Table 6:** The optimal allocation estimation model of the Cr, Pb and Zn concentrations in the soil-wheat system.

Relational Model	Allocation Estimation Model	*r* ^2^	Significance
$BCF=0.1088e^{-0.014C_{s}}$	$C_{w_{Cr}}=0.1088e^{-0.014C_{s_{Cr}}}\times C_{s_{Cr}}$	0.627	P<0.001
$BCF=7E-06C_{s}{}^{3}+7E-05C_{s}{}^{2}-0.0021C_{s}+0.0217$	$C_{w_{Pb}}=7E-06C_{s_{Pb}}{}^{4}+7E-05C_{s_{Pb}}{}^{3}-0.0021C_{s_{Pb}}{}^{2}+0.0217C_{s_{Pb}}$	0.632	P<0.001
$BCF=1.3334e^{-0.016C_{s}}$	$C_{w_{Zn}}=1.3334e^{-0.016C_{s_{Zn}}}\times C_{s_{Zn}}$	0.784	P<0.001

**Table 7:** Error analysis of predicted values for heavy metals in wheat unit: %.

Error	Cr	Pb	Zn
Minimum relative error	0.028	0.951	1.415
Maximum relative error	17.367	57.062	33.336
Average relative error	3.154	11.629	7.789
Relative standard error	0.209	2.664	0.654

## References

[1] Nickel S., Schroder W. (2017). Integrative Evaluation of Data Derived from Biomonitoring and Models Indicating Atmospheric Deposition of Heavy Metals. Environ. Sci. Pollut. Res. Int..

[2] Beattie R.E., Henke W., Davis C., Mottaleb M.A., Campbell J.H., Mcaliley L.R. (2016). Quantitative Analysis of the Extent of Heavy-Metal Contamination in Soils Near Picher, Oklahoma, within the Tar Creek Superfund Site. Chemosphere.

[3] Xing W.Q., Zheng Y.L., Scheckel K.G., Luo Y.M., Li L.P. (2019). Spatial distribution of smelter emission heavy metals. Environ. Monit. Assess..

[4] Guo X.Y., Zhao Q.S. (2011). Ability of soil self-purification for petroleum contaminants—A case study in the third factory of Zhongyuan oilfield. Glob. Geol..

[5] Akpor O.B., Muchie M. (2010). Remediation of heavy metals in drinking water and wastewater treatment systems: Processes and applications. Int. J. Phys. Sci..

[6] Ran J., Wang D.J., Wang C., Zhang G., Zhang H.L. (2016). Heavy Metal Contents, Distribution, and Prediction in a Regional Soil–Wheat System. Sci. Total. Environ..

[7] Adams M., Zhao F., McGrath S., Nicholson F., Chambers B. (2004). Predicting Cadmium Concentrations in Wheat and Barley Grain Using Soil Properties. J. Environ. Qual..

[8] Shi G.L., Zhu S., Bai S.N., Xia Y., Lou L.Q., Cai Q.S. (2015). The Transportation and Accumulation of Arsenic, Cadmium, and Phosphorus in 12 Wheat Cultivars and Their Relationships with Each Other. J. Hazard. Mater..

[9] Baize D., Bellanger L., Tomassone R. (2009). Relationships between Concentrations of Trace Metals in Wheat Grains and Soil. Agron. Sustain. Dev..

[10] Wu J., Norvell W., Hopkins D., Welch R. (2002). Spatial Variability of Grain Cadmium and Soil Characteristics in a Durum Wheat Field. Soil. Sci. Soc. Am. J..

[11] Wang S.Y., Wu W.Y., Liu F., Liao R.K., Hu Y.Q. (2017). Accumulation of Heavy Metals in Soil-Crop Systems: A Review for Wheat and Corn. Environ. Sci. Pollut. Res..

[12] Xiao R., Wang S., Li R., Wang J.J., Zhang Z.Q. (2017). Soil Heavy Metal Contamination and Health Risks Associated with Artisanal Gold Mining in Tongguan, Shaanxi, China. Ecotoxicol. Environ. Saf..

[13] Fang T., Liu G.J., Zhou C.C., Yuan Z.J., Lam P.K.S. (2014). Distribution and Assessment of Pb in the Supergene Environment of The Huainan Coal Mining Area, Anhui, China. Ecotoxicol. Environ. Saf..

[14] Dou Y., Howard K.W.F., Qian H. (2016). Transport Characteristics of Nitrite in a Shallow Sedimentary Aquifer in Northwest China as Determined by a 12-Day Soil Column Experiment. Expo. Health.

[15] Sui H.J., Wu X., Cui Y.S. (2006). Modeling Heavy Metal Movement in Soil: Review and Further Study Directions. Trans. CSAE.

[16] Peng C., Wang M., Chen W. (2016). Modelling Cadmium Contamination in Paddy Soils Under Long-Term Remediation Measures: Model Development and Stochastic Simulations. Environ. Pollut..

[17] Davari M., Homaee M., Rahnemaie R. (2015). An analytical deterministic model for simultaneous phytoremediation of Ni and Cd from contaminated soils. Environ. Sci. Pollut. Res..

[18] Li N., Ren B.Z., Zhou Y.Y., Zhang Y. (2017). Study on the Model of Heavy Metal Migration in Mining Soil. Adv. Environ. Res..

[19] Chen H.Y., Yuan X.Y., Li T.Y., Hu S., Ji J.F., Wang C. (2016). Characteristics of Heavy Metal Transfer and their Influencing Factors in Different Soil–Crop Systems of the Industrialization Region, China. Ecotoxicol. Environ. Saf..

[20] Rezapour S., Atashpaz B., Moghaddam S.S., Damalas C.A. (2019). Heavy Metal Bioavailability and Accumulation in Winter Wheat (Triticum Aestivum L.) Irrigated with Treated Wastewater in Calcareous Soils. Sci. Total. Environ..

[21] Nan Z.R., Li J.J., Zhang J.M., Cheng G.D. (2003). Study on Discrimination of Transfer Control Variables for Heavy Metals in Soil-Root System. Environ. Pollut. Control..

[22] He F., Li R.M., Wang Y., Cao F., Zhang Y.P., Hu Y.H., Wan J.W. (2008). The Study and Application of the Response Relation about Chromium in the Soil-Wheat System in Huanghuai Plain, Henan Province, China. Earth Environ..

[23] Dong J.H. (2008). Distribution of Heavy Metals in Reclaimation Soils and Their Accumulation in Crops. Ph.D. Thesis.

[24] Qiang C., Qin Y., Ding Y., Cao D., Wang F., Zhang M., Han B., Li W.W. (2016). Pollution Characteristics of Heavy Metals in Soils and Wheat Grains in Xuzhou Area. Ecol. Environ. Sci..

[25] Wang Y.J., Ou M.H. (2017). Contents and Distribution of Soil Nutrients and Heavy Metal Elements in Farmlands of Xuzhou. Acta. Pedologica. Sinica..

[26] Shan A.Q., Zhang W., Zhou H.Y. (2016). Pollution Characteristics and Health Risk Assessment of Heavy Metal in Different Functional Zones of Xuzhou. Environ. Eng..

[27] Chen Q., Lu G.C. (1989). Trace Elements and Health.

[28] Zang W.C., Ye J., Tian W., Wang Y.J., Zhao J. (2018). Heavy Metal Pollution and Control.

[29] Liao Z.J. (1989). Pollution Hazard and Migration Transformation of Trace Heavy Metal Elements in the Environment.

[30] Ministry of Ecology and Environment of the People’s Republic of China Environmental Quality Standard for Soils (GB15618-1995). http://www.zbgb.org/2/StandardDetail478530.htm.

[31] Yang G.H., Zhu G.Y., Li H.L., Han X.M., Li J.M., Ma Y.B. (2018). Accumulation and Bioavailability of Heavy Metals in a Soil-Wheat/Maize System with Long-Term Sewage Sludge Amendments. J. Integr. Argic..

[32] Xu C., Zhao T., Chi H.T., Li Q.M., Cheng Y.X., Zhao X.X., Liu W.L., Gao X. (2019). Determination of Eight Kinds of Heavy Metal Elements in Cultivated Soil and the Wheat by Microwave Digestion-ICP-MS method. China Meas. Test.

[33] Li Y.P., Wang S.L., Nan Z.R., Zang F., Sun H.L., Zhang Q., Huang W., Bao L.L. (2019). Accumulation, Fractionation and Health Risk Assessment of Fluoride and Heavy Metals in Soil-Crop Systems in Northwest China. Sci. Total. Environ..

[34] Zhang Y., Yin C.B., Gao S.Z., Cheng L.L., Wu G.S., Guo J.B. (2018). Heavy Metal Accumulation and Health Risk Assessment in Soil-Wheat System Under Different Nitrogen Levels. Sci. Total. Environ..

[35] Ahmadi M., Jorfi S., Azarmansuri A., Jaafarzadeh N., Mahvi A.H., Soltani R.D.C., Akbri H., Akhbarizadeh R. (2017). Zoning of Heavy Metal Concentrations Including Cd, Pb and As in Agricultural Soils of Aghili Plain, Khuzestan Province, Iran. Data Br..

[36] Hakanson L. (1980). An Ecological Risk Index for Aquatic Pollution Control: A Sediment Ecological Approach. Water Res..

[37] Ministry of Health and Standardization Administration of the People’s Republic of China (2005). Hygienic Standard for Grains (GB 2715-2005). http://www.gb688.cn/bzgk/gb/newGbInfo?hcno=3E91E1F7D022C8467BB880CFB1D84E8B.

[38] Ministry of Agricultrure of the People’s Republic of China (2004). Limits of Eight Elements in Cereals, Legume, Tubes, and Its Products (NY 861-2004). http://www.zbgb.org/27/StandardDetail853564.htm.

[39] Huang S.W., Jin J.Y. (2008). Status of Heavy Metals in Agricultural Soils as Affected by Different Patterns of Land Use. Environ. Monit. Assess..

[40] Garcíagonzalo P., Ae P.D.R., Pirredda M., Gismera M.J., Lobo M.C., Pérezsanz A. (2017). Phytoavailability of Cr in Silene Vulgaris: The Role of Soil, Plant Genotype and Bacterial Rhizobiome. Ecotoxicol. Environ. Saf..

[41] Shahid M., Shamshad S., Rafiq M., Khalid S., Bibi I., Niazi N.K., Dumat C., Rashid M.I. (2017). Chromium Speciation, Bioavailability, Uptake, Toxicity and Detoxification in Soil-Plant System: A Review. Chemosphere.

[42] JSCHINA.COM.CN (2016). Precipitation Data in Xuzhou City from January to August Remain High. http://jsnews2.jschina.com.cn/system/2016/09/27/029703286.shtml.

[43] Xuzhou Environmental Protection Agency Environmental Status Bulletin of Xuzhou in 2016. http://hbj.xz.gov.cn/hbj/hjzl/20170605/006001_f75423ec-41fa-43cd-bfb9-605986b6d3f6.htm.

[44] Chen N., Pan Q., Song M.Q., Zhang S.B., Yang Z., Zhang L. (2015). Study on the Heavy Metals Pollution of TSP from Xuzhou. Instrum. Anal. Monit..

[45] Miao X.H. (2018). Analysis and Research on Air Pollution of Xuzhou in November 2017. China Resour. Compr. Util..

[46] Qian T.T., Wu P., Qin Q.Y., Huang Y.N., Wang Y.J., Zhou D.M. (2019). Screening of Wheat Straw Biochars for the Remediation of Soils Polluted with Zn (II) and Cd (II). J. Hazard. Mater..

[47] Yu T., Yang Z.F., Zhong J., Cheng X.B. (2008). Factors Affecting the Geochemical Behavior of Heavy Metal Elements Pb and Cd in Soil. Earth Sci. Front..

[48] Dai W.T., Dong J.H., Di C.L. (2014). Improvement of Identical-Discrepant-Contrary Trend Division in IDC Grey Correlation Analysis. Appl. Math..

[49] Sillers W.S., Fredlund D.G., Zakerzaheh N. (2001). Mathematical Attributes of Some Soil–Water Characteristic Curve Models. Geotech. Geol. Eng..

[50] Champagne C.M., Staenz K., Bannari A., McNairn H., Deguise J.C. (2003). Validation of a Hyperspectral Curve-Fitting Model for the Estimation of Plant Water Content of Agricultural Canopies. Remote. Sens. Environ..

[51] Pan G.X., Chang A.C., Page A.L. (2002). Modeling Transfer and Partitioning of Potentially Toxic Pollutants in Soil-Crop System for Human Food Security. Chinese. J. Appl. Ecol..

